# Exoseketons: A Rehab Tech Consumer's Unexpected March to Action

**DOI:** 10.33137/cpoj.v4i2.37250

**Published:** 2021-09-21

**Authors:** C. Angus

**Affiliations:** 1 Chloe Angus Design, Vancouver, BC, Canada.; 2 Human in Motion Robotics, Vancouver, BC, Canada.

**Keywords:** Disability, Rehabilitation, Ankle Foot Orthotics, Designer, Fashion, Handicap, Exoskeleton

## Abstract

This paper is both a stakeholder perspectives as well as a ‘case study’ describing a journey from sudden disability to participant and investor in exoskeleton design. It tells of my experiences and opinions, as a successful fashion designer, when my life took a drastic turn on becoming paralysed from the waist down over the course of 24 hours, by a spinal tumour. Getting this diagnosis was ‘the worst day in my life’, and it was quickly followed by the ‘second worst’ when, in my determination to walk again, I received Knee Ankle Foot Orthotics (KAFOs) and was shocked to learn that this appeared to be the best technology solution available on the market ‘suitable’ for use in the community. Initial anger at the system for not being better, at the rehab team for their complacency, and at myself for allowing a feeling of helplessness to take over led to questions such as: what does this say about our society? and what are we all willing to accept, for ourselves and others? This is professional opinion and an essay about how we see ourselves and how others see us. The journey from pre-injury ‘consumer’ to post-injury ‘disabled’ person and learning that being labeled ‘disabled’ leads to the additional handicap of the narrow vision of “cost” taken by the mobility industry where innovative ideas are stripped down to the bare minimum with the excuse that patients are “lucky” to have what they have been “given”. Grappling with these labels and inequities and seeking a better outcome, I became an integral team member of an exoskeleton development team, leading to the design of The Next Generation Exoskeleton! This is MY story, the story of Chloe Angus. It is the story of inclusive, user focused design and is a call to include and respect the end users of all assistive device technology design early in the design process and it is being told from the perspective of a person having experience and success in the world of business.

## INTRODUCTION

Think about getting old. We all get older and, with aging, come arthritic knees, broken hips and other challenges. Mobility challenges eventually become everybody's problem and the solutions that are out there to help us move are like stone aged ‘sticks” (canes) and stone wheels (wheelchairs).

But, times have changed and we can create something infinitely better, something revolutionary by using technology to move us beyond our physical limitations. This is not the way of the future. It is here now. Cutting-edge materials, 3D Printing, Artificial Intelligence (AI), and advanced engineering can be used to create smart prosthetics, stylish orthotics, and things as futuristic as wearable robotic suits. It is technology innovation and it is my story!

### My name is Chloë Angus

I am a fashion designer and mobility activist. I am the end user of ‘stone age’ mobility devices.

In 2015, I had achieved the life I had always dreamt of: strolling down runways and red carpets, meeting movie stars, media personalities, politicians, and princesses. Drinking bubbly for breakfast and wearing ball gowns to picnics. Married for 15 years and still madly in love, my husband Gabe and I had worked hard to carve out the life we wanted. I had no idea the drastic turn life was about to take.

On a typical June Sunday I went for a run and limped home with my right hip aching. A few hours later the toes on my right foot start tingling, going numb. I drove myself to Vancouver General Hospital thinking: Four hours to get through Emergency and I'll be back, catching up at work.

Unfortunately, that tingling crept up both legs and I watched helplessly as my legs stop working. 24 hours later one of the best doctors in Canada told me I was paralyzed from the waist down due to a rare benign tumor in my spinal cord and I heard him say I was probably born with it. I also heard the words “loss”, “function”, “acceptance”. Then that doctor, with his perfect hair and calm voice, looked me directly in the eyes and said “You will never walk again.” Struggling to keep my composure, I focused on the red Ferrari lanyard around his neck, thinking this isn't possible. I was running yesterday. I drove myself to this hospital! And inexplicably I thought: How many injuries did it take to own that Ferrari? And, how often does he say the words ‘You Will Never Walk Again?’ I felt the blood drain from my face, my heart tighten, and I began to envision my life in a wheelchair.

### A Conversation

In one day, I went from being a busy entrepreneur who “ran” a fashion design company to not being able to get out of a chair. Over the next few weeks reality set in. I became aware of the serious secondary health complications of living in a wheelchair. Muscles atrophy, bowel and bladder complications, circulation problems, pressure sores and bone density loss from not weight-bearing.

Then there was the extreme pain, in my case, caused by sitting too long. When my doctors offered me a fentanyl patch program for the pain I knew the world needs a better solution than the wheelchair I had. It was simply unacceptable and all of us deserve better options. This is the passion that drives me as an innovator.

The circumstances leading to my losing mobility are rare, but mobility loss is more common that most people imagine. MS, Parkinson's, Stroke or Brain injury, along with something as simple as aging can result in the limitation or loss of mobility. So can a car accident on the way to work, or a fall on the ski hill. Retirement Homes and Rehabilitation Centers are full of wheelchairs and walkers. At some point in life, mobility becomes everybody's problem.

### Darkest Night and the Fight to Keep Moving

That night after my doctor told me “You'll NEVER walk again.” (my emphasis), lying alone in my hospital room, I replayed the doctor's words and made two important decisions.

First: I would refuse to give up hope! Being hopeful is not denying your reality. Being hopeful is believing that nothing is impossible. Armed with hope, the second decision was easy: I will not take this sitting down. I will fight for mobility. I will walk again!

Desperate to get back into my studio, because the fashion cycle waits for no one, I got on Google and found an article in Popular Science Magazine, about a new technology called an exoskeleton that could help paralyzed people walk again. It was a wearable robotic suit, like in the movie Ironman. The next morning, I said to my husband “Honey! Just order me one of this off eBay and I will be back at work next week!” Unfortunately, I learned, you can't buy one on eBay. Several months later at the rehabilitation centre, and after much advocating for myself, I finally was allowed to try an exoskeleton.

I thought: HA! That doctor had said “YOU will NEVER walk again”. He was WRONG! He may have meant that I will never walk like I once did, but there are many ways to walk. What a feeling. The first time I put on the exoskeleton and walked again, after so many doctors told me I would not, was vindicating and opened my eyes to what was possible with technology. Euphoric, I was ready to walk right out of the rehabilitation center.

However, I quickly learned that exoskeletons, and the orthotics that go with them, were extremely limited. The device I used only allowed me to move forward in a rudimentary robotic way, not allowing for natural walking gait or balance. It also it required arm crutches and an attendant to use it. It could not be used independently or go downstairs or up curbs. The ‘orthotics’, where hard straps that pinched. Visually I looked as awkward and as uncomfortable as I felt. This was clearly not a robotic suit as shown in the movies.

Still, the technology was amazing. It just needed to be improved. This was in 2015 and, at that time, I had no idea I would become an advocate for exoskeletons, collaborator with a tech company, or a designer of orthotics.

### Going Back in Time

My journey did not end with my rehabilitation or first use of an exoskeleton. Weeks into my rehabilitation, I was given the diagnosis of “complete” spinal cord injury with the prognosis of “no chance of recovery”. After being able to use the exoskeleton at the rehab centre, as a result of my persistent requests and self-advocacy, I was told my time was up and the cost of my continuing to use the exoskeleton was not “worth” it. I was once again reminded that I would not recover and that I would have to learn to live my life in a wheelchair.

**Figure 1:**
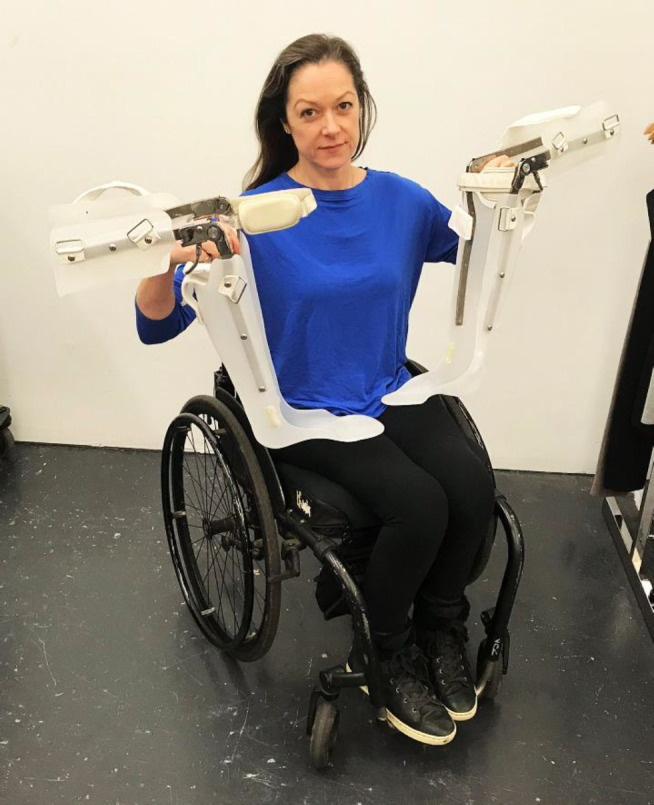
The second lowest day of my life.

When I persisted and told my physio team I wanted to walk again they suggested I get leg braces called KAFO's. I was made a pair, at the cost of $11,000, and the day I received them was the lowest day in my life after the day I was told I would never walk again. They looked like something out of a history book or the Forest Gump movie (**Figure 1**).

The day after I walked in a wearable robotic suit from the future, the rehab centre's exoskeleton, I found myself in the basement of a hospital being fit for leg braces made of metal rods and leather straps that looked like they came out of a history book. The contradiction made me second guess my own sanity. Was I in 1950? How did I get here and how do I get back to the future?

### Re-commitment to Hope: Refusing to Accept What I am “Given” and Believing Ironman is Possible

Rolling out of the orthotics dungeon, with tears in my eyes, I got mad. Mad at the system for not being better, mad at my rehab team for their complacency, and mad at myself for the feeling of helplessness that I had allowed to take over. Thoughts about acceptance kept coming back to me: What are we all willing to accept, for ourselves and others?

Mobility becomes an issue for all of us at some point in all our lives. Mobility deserves advanced solutions, whether it is a simple insole that allows you to keep running well into your 90's or a full body exoskeleton being worn by 10-year-old tetraplegic to school. Society must believe in something better; believe that “Ironman” is possible. But how does one shift an entire industry that seems to have become too complacent?

### Shifting Perspectives to the Consumer

Typically, products and solutions for disabled people are designed and developed by able bodied people. Worse, I learned, they are often designed to meet the needs of able-bodied people: Hospitals, Rehab Centres, and Insurance Providers, those who pay the bills, are their target market. It is these bodies that typically dictate what products are made available in the mobility industry and not the people who need and use the products themselves. Even worse, the actual customer, who needs the device and finds themselves in a desperate situation is made to feel they should be grateful for what they are given, even when it is often not what they need or want.

The general population, including my ‘designer self pre-injury’, do not know anyone in a wheelchair. That changed when I became a wheelchair user and, after just one day, it was clear my needs where not being met. Almost overnight, I became an expert in a new field: the field of mobility. We can't address what we don't know, this is why it is so important for developers in all fields engage with those lived experts.^1^

My injury attached the label of ‘disabled’ on me. Before that I was a ‘consumer’. As a consumer there were millions of choices offered to me. From colour to cost, brands catered to my every whim. When I shop for what I “need” now, as a ‘disabled person’ what I want is no longer offered to me as a choice. I am no longer seen as a ‘consumer’ but only as a “end-user with a disability”.

This narrow vision of “cost” taken by the mobility industry is dictated by Hospitals, Rehab Centres, and Insurance providers. Innovative ideas are stripped down to the bare minimum with the excuse that patients are “lucky” to have what they have been “given”. This narrow view of ‘cost’ also does not appear to consider the long-term cost of the secondary health complications a lack of mobility inflicts on individuals and the system. And it restricts those trying to come up with new solutions. If we think of cost before all else, we would not have cars, let alone people walking on the moon.

**Figure 2:**
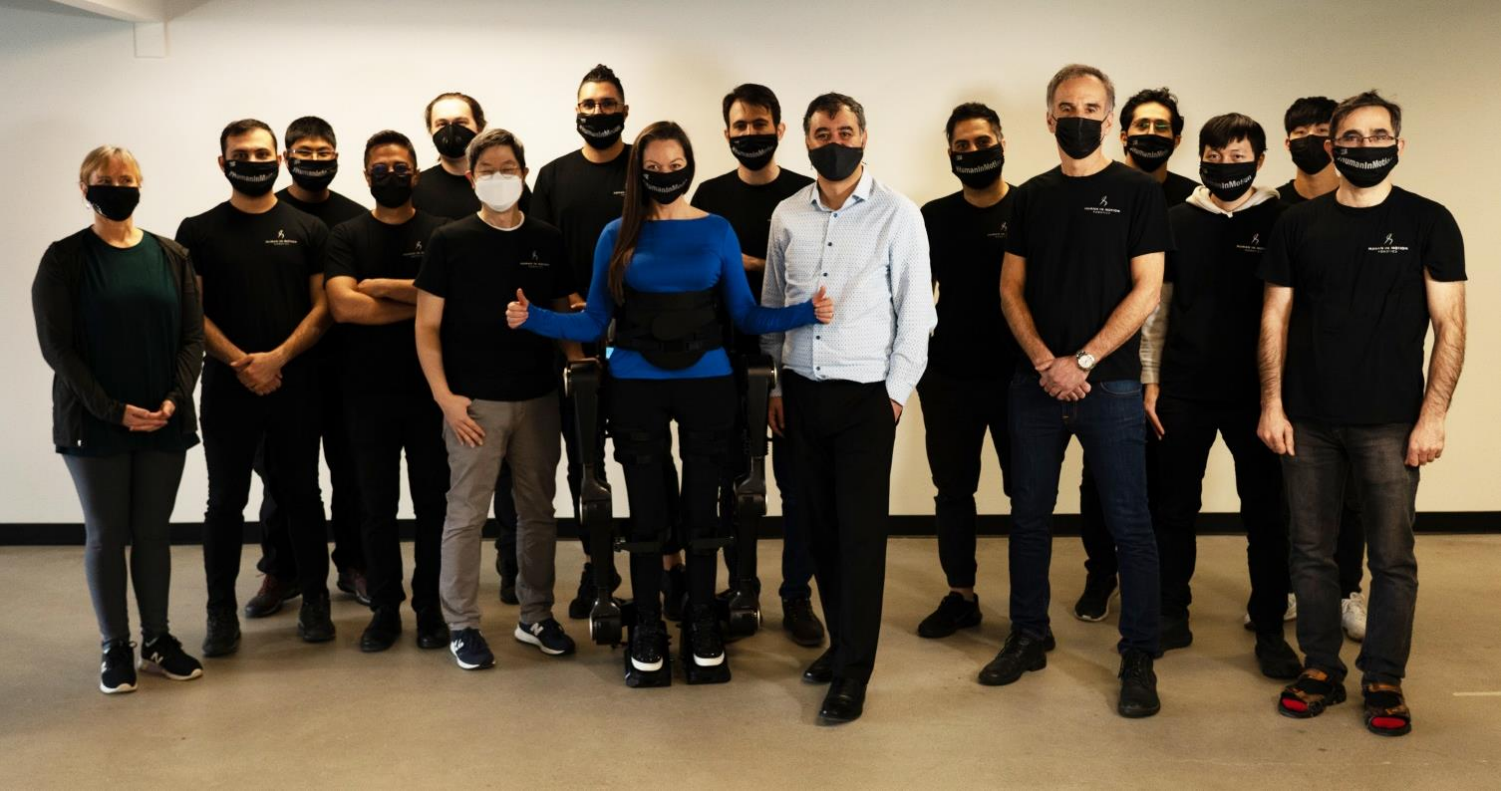
The Simon Fraser University/Human in Motion Team. Persons in image have given informed consent to publication.

As a CEO, I am well aware that costs are important factor when running a successful business, but ultimately the mobility industry needs a consumer-focused perspective shift: from patient to client if it is to grow, have market support, and ultimately happy consumers. It is always people and community, not governments, that make real change. Fortunately, I found an academic team that had a similar hopeful vision for the future as I did (**Figure 2**).

### The Road to Hope

The experience of walking again, with the help of an exoskeleton was my first step to a new, technology assisted, future. No longer having access to the Rehab Centre exoskeleton and having found I could not order one on Amazon, I started searching for access to this technology.

This led me to two engineering professors at Simon Fraser University in Surrey, BC, Canada, Dr. Siamak Arzanpour and Dr. Ed Park. In collaboration with them, we started Human In Motion Robotics (HMR) and are working with a growing team of engineers, researchers, students, industry leaders, and investors to build the world's most advanced exoskeleton for rehabilitation and personal use.

The technology being developed is a radical improvement to the current technology, capable of advanced articulation and superior range of motion, allowing for natural walking, self-balancing capabilities, and independent use.^2^ It will allow me, and 80 million others that live with motion disabilities, to walk back into our lives with dignity and independence. The development team is working all aspects – from the technical, to orthotic fit, to focusing on practical client needs and aspirations in addition to accessibly–with the goal being that the next generation of the HMR exoskeleton will be available for purchase online and will meet the needs and wants of customers. As a designer of fashion, and now wearable and exoskeleton prototype in the lab, I envision a sleek, stylish, robotic suit - in custom colours, making it “cool” to wear. People wearing our robotic suit will feel confident in the boardroom, at a cocktail party, or in their seniors' living home.

Company leadership like that at HMR are to be commended by actively including those with lived experience. This approach is instrumental in making the necessary shift in the industry and creating successful markets for developed products.

### Hope Regained: My Brightest Day

**Figure 3:**
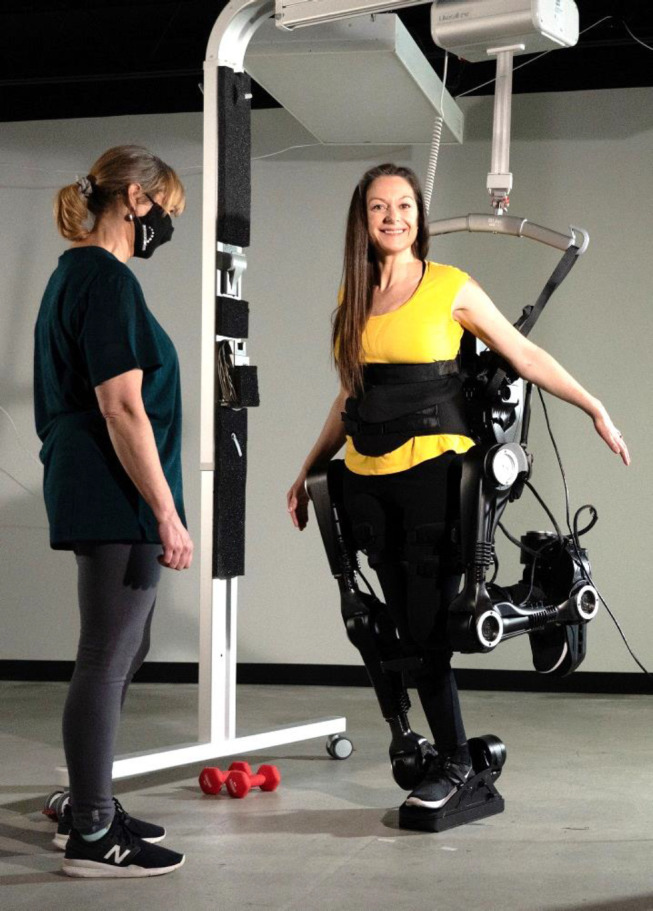
Myself, wearing the first-generation prototype of the Human in Motion Exoskeleton, standing on one leg independently. The harness is there just for safety, it is not supporting me.

What I have described above are lofty design aspirations. My darkest day had been the night in hospital where I kept thinking about the words “You will never walk again”. Many months later, and after much learning, I had one of my best, hopeful days–June 21st 2019. The day started with a high-profile client putting on a beautiful ball gown I designed with perfect fit and proportion, a vision of red silk and showcasing the skills I had developed in my years as a fashion designer. Later that same day, I tried on a new “outfit” of my own, that I had also helped design, The Next Generation Exoskeleton (**Figure 3**). I walked independently, no attendant, no arm crutches. That was a bright day. I felt successful back in my career and – without any help or aids– I WALKED AGAIN!

## CONCLUSION

### Walking Forward Together

My journey from ballgowns to wearable robotic suits has come full circle. Emotionally I feel hopeful, inspired and truly alive. Today, I am working on growing awareness around the issues of mobility disability and am working with, and investing in, a team of advocates and innovators– people who will take a stand for advancing technology until all mobility challenged people can ‘Stand for Themselves’!

Take a minute to envision the future of your own mobility. Imagine if you become mobility impaired instead of being limited to a wheelchair, you now have options. Options that will allow you to walk anywhere. You can stand to deliver a speech. With your exoskeleton, you will feel equal again, standing eye to eye when you shake hands. You can hug your loved ones to your chest, maybe even walk down the aisle. Options that provide both physical mobility and access to the world around you.

Canes, walkers, and wheelchairs are not enough. We walked on the moon 50 years ago! We have the technology; all we must do is apply it. Insurance and Healthcare providers must get on board and those who can, should invest in advanced mobility technology for all.

Talk to your friends, family, and government about the need for advanced mobility. We can all be advocates. In our lifetime, people of all ages and abilities will walk again!

## CALL TO ACTION

- Don't make assumptions about individuals with disability. The responsibility for this relies not only on the general public and health care providers but also on tech developers and funders of health tech. Get your hands dirty, get to know people with disabilities and what their aspirations are.
- For the funders of health tech, calculate the quality and dignity of the experience of life into your value calculations – just like you are now taking Social Justice, Gender and Environmental (SGE) criteria into account.
- Don't make assumptions about what is possible. Look at what has been done like sending someone to the moon and aim big. Invest in a technologically improved future. The responsibility for this relies both on health and rehabilitation professionals and biomedical engineers.
- Advocate for the right to return to an active, integrated lifestyle and the funding investment required to both develop that necessary technology and the ability to get it to market and accessible to those who require it. The responsibility for this lies with researchers, funders, insurers and all disability advocates.
- And understand that accessibility not only means that a person regains mobility and independence but that the technology required is easily accessible, from funding to on-line business models. Business models in all other sectors are changing, rehab technology business models have to change with them.

## DECLARATION OF CONFLICTING INTERESTS

Chloe Angus is a shareholder in Human in Motion Research and is a member of the team developing a novel exoskeleton design.

## SOURCES OF SUPPORT

The Human in Motion exoskeleton is being carried out with support from the NSERC Engage Program, the Praxis Spinal Cord Institute Accelerate Program, and the Canadian SR&ED Program.
